# Triglyceride Induced Metabolic Inflammation: Potential Connection of Insulin Resistance and Recurrent Pregnancy Loss

**DOI:** 10.3389/fendo.2021.621845

**Published:** 2021-04-15

**Authors:** Yongjie Liu, Mengyang Du, Yuexin Gan, Shihua Bao, Liping Feng, Jun Zhang

**Affiliations:** ^1^ Ministry of Education and Shanghai Key Laboratory of Children’s Environmental Health, Xinhua Hospital, Shanghai Jiao Tong University School of Medicine, Shanghai, China; ^2^ Department of Reproductive Immunology, Shanghai First Maternity and Infant Hospital, Tongji University School of Medicine, Shanghai, China; ^3^ Department of Obstetrics and Gynecology, Duke University School of Medicine, Durham, NC, United States

**Keywords:** triglyceride, CD3^+^CD4^+^/CD3^+^CD8^+^ ratio, insulin resistance, recurrent pregnancy loss, insulin sensitivity

## Abstract

The underlying correlative mechanisms between Insulin resistance (IR) and recurrent pregnancy loss (RPL) in patients without polycystic ovarian syndrome (PCOS) remain inconclusive. To investigate the association between triglyceride (TG) levels, lymphocyte subsets, and IR in RPL patients without PCOS and obesity. Eighty-nine subjects with an unexplained RPL, independent of PCOS/obesity were enrolled in this study. A 75-g oral glucose tolerance test was performed on each subject with plasma tested for glucose and insulin. The fasting venous blood of all subjects was collected for routine clinical chemistry analysis. Lymphocyte subsets were analyzed by four-color flow cytometry. As a result, TG levels were significantly elevated in RPL patients with IR compared to those without IR. Pearson linear correlation model and receiver operating characteristic (ROC) curve analyses revealed a significant positive association between TG and HOMA-IR index value. In multiple logistic regression analysis, TG was significantly associated with the risk of hyperinsulinemia and increased CD3^+^CD4^+^/CD3^+^CD8^+^ ratio which was significantly negatively correlated with disposition index (DI30) and DI120, indicators for insulin sensitivity. In addition, DI30 and DI120 were significantly decreased in the higher CD3^+^CD4^+^/CD3^+^CD8^+^ group. Our findings showed that the elevated TG and altered immune responses in RPL patients with IR are independent of PCOS and obesity, and could be used as an indicator of IR in RPL patients. These results contribute to the understanding of the pathophysiology of IR in RPL for potential prevention and therapeutic targets.

## Introduction

Miscarriage, the most common complication of pregnancy, is the spontaneous loss of a conceptus before 20 weeks’ gestation, and it affects approximately 15% of reproductive-age women recognized clinically (1). There are two types of miscarriage: sporadic and recurrent. Recurrent pregnancy loss (RPL), defined as three or more consecutive pregnancy losses, occurs in approximately 1% of childbearing couples (2). Actually, many clinicians define RPL as two or more losses, due to the recurrence rate is similar to that after three losses, and it affects around 1% to 5% of couples (2–4). Historically, RPL has been attributed to either genetic, structural, infective, endocrine, immune, or unexplained causes (2, 5–8). Endocrine disturbances have been postulated as an important cause of RPL, which widens the scope of investigation.

In recent years, researchers have focused on the connection between polycystic ovarian syndrome (PCOS), insulin resistance (IR), and RPL (9, 10). About 70% of women with PCOS have IR (11), and spontaneous pregnancy loss occurs in 40% women with PCOS (12). Data from clinical trials also showed that IR is common in women with RPL (13–16), and has been associated with an increased rate of pregnancy loss in PCOS patients (17). In fact, there are many RPL subjects with IR who do not have PCOS and/or obesity. It has been reported that non-obese Asians (BMI < 25 kg/m^2^) can readily develop metabolic abnormalities (18, 19). Tamura et al., also reported that impaired insulin clearance and hyperinsulinemia could occur in apparently healthy subjects (20). Studies on the underlying correlative mechanisms between IR and RPL in patients without PCOS remain inconclusive. Guidelines from the Endocrine Society commonly recommend using metformin for women with IR and glucose intolerance due to its functional role on activating the insulin receptor and increasing glucose uptake. However, a randomized double-blind study also indicated that metformin has no major effects on glucose homeostasis in pregnant women with PCOS (21, 22), which indicates there are other underlying causes of IR in RPL patients beyond abnormal glucose metabolism. Therefore, it is imperative to understand the pathophysiology of IR in RPL for potential prevention and therapeutic targets.

Insulin resistance is characterized by a decreased responsiveness of insulin sensitivity and supraphysiological levels of insulin. It has been observed that a chronic inflammatory state may contribute to the development of IR (23, 24). Dysregulation of fat metabolism, characterized by significantly increased lipid accumulations, and triglycerides (TG) level, are known to induce low grade inflammation (25, 26). It was reported that the level of LDL cholesterol increases, whereas that of HDL cholesterol decreases in PCOS (27). Furthermore, elevated TG/HDL cholesterol ratio was significantly associated with IR and could be used as a predictor of IR in women with PCOS (28). However, the link between TG levels, inflammation, and IR in women without PCOS is unclear and need further study. It’s worth noting that the T cell lymphocytes play a pivotal role in regulating the effector stage of the immune response, and increased lymphocyte proliferation is associated with an inflammatory response (29–31). An increased ratio of CD4^+^/CD8^+^ lymphocytes was found in lipopolysaccharide (LPS) challenged pigs (32). IR was reported to be related to the abnormal percentage of lymphocyte subsets in RPL (33, 34). A recent study revealed that a decrease of regulatory B cells (CD19^+^CD24^hi^CD38^hi^) in peripheral blood is associated with IR, and can increase the risk of postprandial hyperinsulinemia (35). Yan et al. also demonstrated that IR was associated with cellular immune abnormalities in RPL patients (34).

Taken together, we hypothesized that cellular immune abnormalities and/or abnormal fat metabolism might contribute to the pathophysiology of IR in RPL subjects without PCOS. To test our hypothesis, we investigated the relationship between fat metabolism and immune cell status in peripheral blood and homeostasis model assessment for insulin resistance (HOMA-IR) parameters in an RPL cohort with/without IR, independent of PCOS/obesity.

## Materials and Methods

### Participant Enrolment

**Figure 1:**
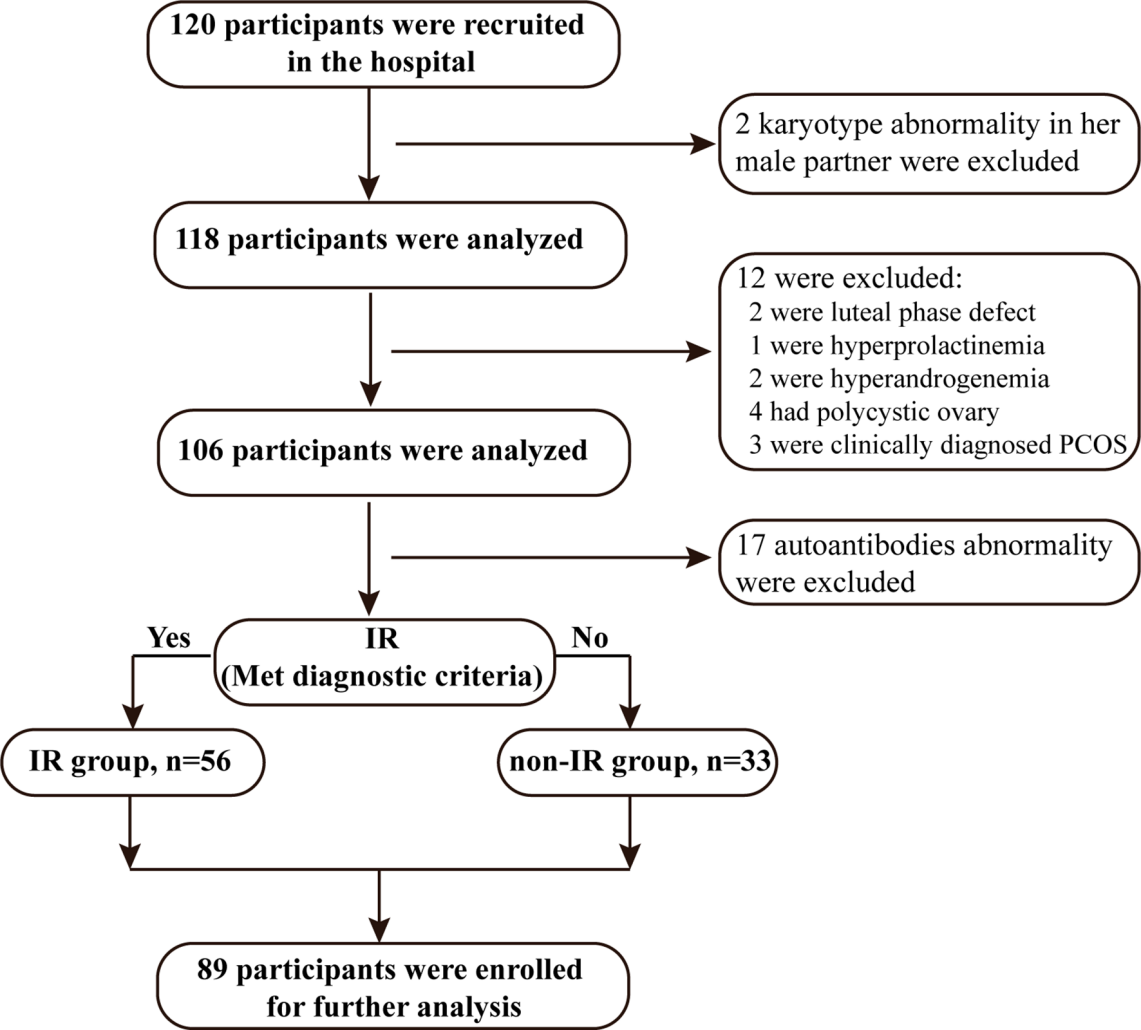
Flow chart for the exclusion in this study.

Women were recruited at the first visit and were eligible for enrollment in this study if: (i) they were age less than 35 years with a body-mass index (BMI) no more than 27.9 kg/m^2^ (non-obesity reference value standards of BMI of Chinese), and (ii) had regular ovulatory menstrual cycles and were actively trying to conceive with a history of unexplained RPL (two or more consecutive pregnancy losses before 20 weeks of gestation). The diagnosis of pregnancy loss was confirmed by ultrasound. (iii) The current male partner had normal semen testing by computer-assisted analysis. Age criterion was applied because the likelihood of pregnancy loss, due to chromosomal abnormalities is higher in older women. Subjects with a verifiable cause of miscarriage were excluded (**Figure 1**). The exclusion criteria were: (i) abnormality of the uterus such as uterine septae and bicornuate uterus; (ii) karyotype abnormality in either the parents or the embryo. Karyotyping abnormity was defined as abnormal chromosome number (such as trisomy, polyploidy, and monosomy X) and abnormal chromosome structure (either a balanced reciprocal translocation or a Robertsonian translocation). (iii) luteal phase defect (diagnosed by combining basal body temperature and serum progesterone levels < 10 ng/mL) (36, 37), hyperprolactinemia (non-pregnant serum prolactin levels > 30 ng/mL), or hyperandrogenemia (serum testosterone levels > 0.7 ng/mL); (iv) with PCOS which was diagnosed by the presence of all following criteria: (menstrual irregularities, polycystic ovarian morphology (PCOM), and/or hyperandrogenemia). Moreover, women diagnosed with non-PCOS with hyperandrogenemia, ovulatory dysfunction, or PCOM are excluded for further analyses; (v) presence of autoantibodies including antinuclear antibodies (ANA), anticardiolipin antibodies (ACL), anti-Beta 2 glycoprotein and extractable nuclear antigens antibodies associated with systemic lupus erythematosus (SLE). The study protocol was approved by the Ethics Committees of Shanghai First Maternity and Infant Hospital (KS1812). Eighty-nine participants were recruited at the Department of Reproductive Immunology, Shanghai First Maternity and Infant Hospital between April 2018 and March 2019. Informed consent was obtained from all participants.

### Oral Glucose Tolerance Test and Insulin Releasing Test

A 75 g oral glucose tolerance test (OGTT) was performed on each subject with plasma tested for glucose and insulin in a fasting state. Glucose levels were measured by a glucose oxidase-based assay (Roche Diagnostics GmbH, Mannheim, Germany). Insulin levels were measured by enzyme immunoassay (Roche Diagnostics GmbH, Mannheim, Germany). The plasma glucose levels at time points 0, 30, 60, 120, and 180 min were designated as Glu0, Glu30, Glu60, Glu120, and Glu180, and the insulin level at these time points were designated as Ins0, Ins30, Ins60, Ins120, and Ins180, respectively. In addition, the mean glucose and insulin levels during OGTT were designated as Glu_mean_ and Ins_mean_, respectively.

### Diagnosis

In this study, IR was diagnosed by the following indicators: 1) impaired glucose tolerance (IGT) or diabetic (38); 2) fasting insulin levels (FINS) ≥ 15 mIU/L (39); 3) HOMA-IR = Ins0 (µIU/ml) × Glu0 (mmol/L)/22.5 > 2.5 (40); and 4) normal FINS, but Ins180 level cannot be back to the normal level (41). The area under the curve (AUC) for glucose and insulin (GluAUC and InsAUC) was determined using the insulin secretion curve adjusted for glucose. The islet function and sensitivity index included the following: (i) a homeostasis model assessment of β cell function (HOMA-β) = [20 × Ins0 (µIU/ml)]/[Glu0 (mmol/L) − 3.5]; (ii) the early insulin secretion index (ΔI30/ΔG30) = [(Ins30 − Ins0) (µIU/ml)]/[(Glu30 − Glu0) (mmol/L)]; (iii) AUC for early-phase insulin secretion (InsAUC30/GluAUC30) = [(Ins0 + Ins30) (pmol/L)]/[(Glu0 + Glu30) (mmol/L)]; (iv) AUC for total insulin secretion (InsAUC120/GluAUC120) = [(Ins0+4×Ins30+3×Ins120) (pmol/L)]/[(Glu0+4×Glu30+ 3×Glu120) (mmol/L)]; (v) the Matsuda insulin sensitivity index (ISI_M_) = 10,000/[(Glu0 (mg/dl) × Ins0 (µIU/ml) × Glu_mean_ (mg/dl) × Ins_mean_ (uIU/ml)]^0.5^; (vi) the disposition index (DI) = HOMA−β/HOMA-IR, representing an adjusted insulin sensitivity according to HOMA-IR; vii) the early-phase disposition index (DI30) = InsAUC30/GluAUC30 × ISI_M_ and the total disposition index (DI120) = InsAUC120/GluAUC120 × ISI_M_.

### Blood Chemistry Assay

The morning fasting venous blood of all subjects was collected for routine clinical chemistry analysis. Serum lipid content, including total cholesterol (TCH), triglyceride (TG), and homocysteine (HCY) was measured using an enzymatic colorimetric assay (Beckman Coulter Inc., Brea, USA). Serum levels of insulin, in addition to thyroid function, including thyrotropin (TSH), free triiodothyronin (FT3), and free thyroxine (FT4) was measured by enzyme immunoassay (Roche Diagnostics GmbH, Mannheim, Germany).

### Flow Cytometry Analysis for Lymphocyte Subsets

The lymphocyte subsets, including CD3^+^, CD3^+^CD4^+^, CD3^+^CD8^+^, CD19^+^, and CD16^+^CD56^+^ (NK) cells were analyzed by four-color flow cytometry. Venous blood was drawn into EDTA anticoagulant tubes and then 200 µL of the fresh whole blood samples were stained with monoclonal antibodies conjugated with fluorochromes fluorescein isothiocyanate [FITC], phycoerythrin [PE], PE cyanine 5 [PE-Cy 5], and allophycocyanin [APC] and incubated for 20 min in the dark at room temperature. Erythrocytes were lysed by FACS lysing solution (BD Biosciences) and then the blood sample was washed with PBS twice prior to flow cytometric analysis. A FACSCalibur flow cytometer with multiset software (BD Biosciences) was used to analyze the levels of subpopulations.

### Statistical Analysis

All data are shown as means ± standard deviation (SD), and statistically analyzed using the SPSS 22.0 software package. Comparisons of continuous variables with normal distribution between the groups were performed using Student’s t-test, and Chi-square test was performed for categorical data analysis. Pearson’s correlation test was used to analyze the correlation between lipid profiles, the lymphocyte subsets, and insulin resistance by regarding as continuous variables. Multiple-factor regression analysis was performed for assessing the association between TG levels, hyperinsulinemia, and CD3^+^CD4^+^/CD3^+^CD8^+^ ratio. *P* < 0.05 was considered statistically significant.

## Results

### Characteristics of Participants.

According to the standard of IR, of the 89 RPL women, 56 participants were diagnosed with insulin resistance and defined as the IR group, and the remaining 33 were the non-IR group. The general characteristics of the study participants are presented in **Table 1**, which include the following epidemiological factors: age, BMI, education level, smoking status, alcohol-drinking status, reproductive tract virus infection, antibodies screen and medical history. The RPL women with IR exhibited significantly higher BMI compared with the non-IR group although all subjects’ BMI were in a healthy weight range. No significant differences were observed regarding other characteristics.

**Table 1:** Characteristics of participants in this study.

Variables	Non-IR (n = 33)	IR (n = 56)	*P-value*
Age (years)^a^	30.5 ± 4.8	30.6 ± 3.4	0.95
Body mass index (BMI, kg/m^2^)	20.9 ± 2.0	22.2 ± 2.6	0.009*
<18.5	6 (18) ^b^	5 (9)	0.34
18.5 ≤ BMI < 24	24 (73)	37 (66)	0.68
24 ≤ BMI < 28	3 (9)	14 (25)	0.12
Waist circumstance (cm)	66.0 ± 2.2	65.9 ± 2.6	0.92
Education			
Illiterate	0 (0)	0 (0)	NE^c^
High school or lower	7 (21)	9 (16)	0.75
College	23 (70)	45 (80)	0.38
Postgraduate or higher	3 (9)	2 (4)	0.54
History of smoking	0 (0)	0 (0)	NE
History of drinking	0 (0)	0 (0)	NE
History of diabetes	0 (0)	2 (4)	0.72
History of hypertension	0 (0)	3 (5)	0.46
Virus			
HIV+	0 (0)	0 (0)	NE
HPV+	1 (3)	0 (0)	0.79
Syphilis	0 (0)	0 (0)	NE
Antibody			
Anticardiolipin antibody (IgA, IgM, IgG)	0 (0)	0 (0)	NE
Anti DNA antibody (single- and double- stranded)	0 (0)	0 (0)	NE
Anti ENA (extractable nuclear antigen, 7 subtypes) antibody	0 (0)	0 (0)	NE
Anti-beta 2 glycoprotein antibody (Ig, IgG)	0 (0)	0 (0)	NE
Irregular antibody	0 (0)	0 (0)	NE
Medical history			
Endometriosis	0 (0)	0 (0)	NE
Uterine fibroids	2 (6)	0 (0)	0.26
Endometrial polyps	0 (0)	0 (0)	NE
Intrauterine adhesion	2 (6)	2 (4)	0.99
Ovarian cysts	0 (0)	0(0)	NE
Pelvic inflammation	0 (0)	0 (0)	NE

^a.^Data are presented as mean ± SD. ^b.^Data are presented as n (%). ^c.^NE, not estimable (due to nullity of category in both groups). Pelvic inflammation means Pelvic Inflammatory Diseases, including endometritis, salpingitis, and chronic pelvic inflammation.

### Comparison of β-Cell Function and Insulin Sensitivity Between IR and Non-IR Group

**Figure 2:**
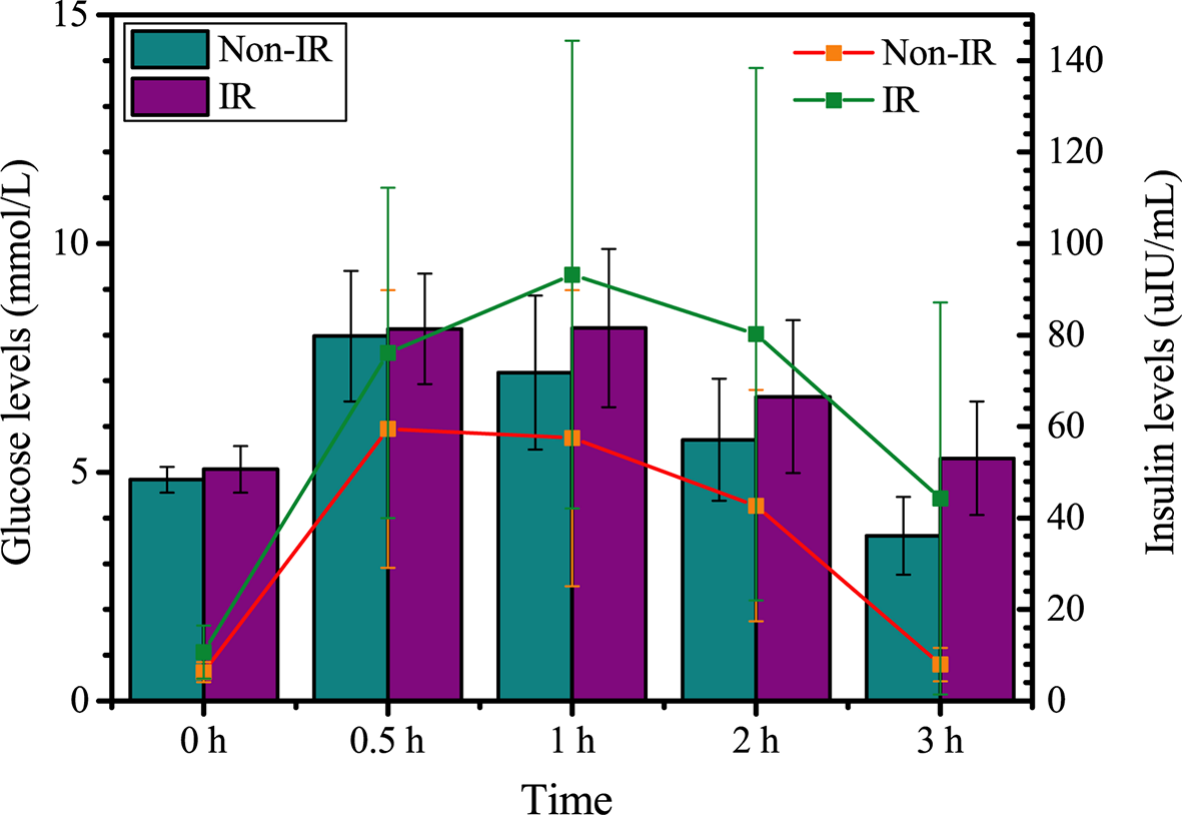
Glucose and insulin levels between insulin resistance group (IR) and non-insulin resistance group (Non-IR).

As shown in **Table 2**, HOMA-β was significantly higher in the IR group than the non-IR group. Patients with IR exhibited higher values of InsAUC30/GluAUC30 and InsAUC120/GluAUC120, which reflected higher early-phase insulin release and total insulin release, respectively, as opposed to the controls. In addition, RPL women with IR showed a significant decrease in Matsuda index, DI, DI30, and DI120 during OGTT, which indicates the reduced insulin sensitivity. Moreover, patients with IR manifested significantly higher glucose levels at 0, 60, 120, 180 min during OGTT in comparison with the controls, while the insulin levels were markedly higher during the entire period of the OGTT compared to the non-IR group (**Figure 2**).

**Table 2:** Comparison of β-cell function and insulin sensitivity, lipid profiles, and lymphocyte subsets of RPL patient with IR and non-IR.

		Non-IR (n = 33)	IR (n = 56)	*P-*value
β-cell function and insulin sensitivity	HOMA-IR	1.3 ± 0.5	2.6 ± 1.5	<0.0001*
	HOMA-β	92.5 ± 33.4	144.2 ± 69.2	0.0002*
	ΔI30/ΔG30	20.7 ± 18.1	21.6 ± 10.7	0.78
	InsAUC30/GluAUC30	36.2 ± 16.8	46.6 ± 21.1	0.03*
	InsAUC120/GluAUC120	49.8 ± 21.0	67.4 ± 30.0	0.007*
	Matsuda index (ISI_M_)	8.0 ± 3.0	4.9 ± 2.3	<0.0001*
	DI	75.7 ± 20.4	62.0 ± 20.1	0.005*
	DI30	261.0 ± 96.1	194.7 ± 72.7	0.0009*
	DI120	358.1 ± 117.2	275.8 ± 81.9	0.0005*
Lipid profiles	TCH (mmol/L)	4.4 ± 0.7	4.4 ± 0.9	0.97
	TG (mmol/L)	0.8 ± 0.4	1.1 ± 0.6	0.025*
	LDL-C (mmol/L)	2.8 ± 0.3	2.9 ± 0.4	0.35
	HDL-C (mmol/L)	1.3 ± 0.3	1.4 ± 0.4	0.17
	HCY (µmol/L)	9.5 ± 2.8	8.6 ± 2.3	0.20
	TSH (mIU/L)	1.8 ± 0.9	2.2 ± 1.0	0.11
	FT3 (pmol/L)	5.0 ± 0.4	5.0 ± 0.6	0.97
	FT4 (pmol/L)	16.2 ± 2.1	17.2 ± 2.3	0.12
Lymphocyte subsets	CD3^+^ cells (%)	70.1 ± 9.7	67.6 ± 8.4	0.22
	CD3^+^CD4^+^ cells (%)	36.1 ± 6.9	34.9 ± 6.4	0.43
	CD3^+^CD8^+^ cells (%)	28.1 ± 7.6	26.9 ± 6.0	0.39
	CD3^+^CD4^+^/CD3^+^CD8^+^ cell Ratio	1.4 ± 0.5	1.4 ± 0.4	0.89
	CD16^+^CD56^+^ cells (%)	16.6 ± 9.1	18.7 ± 8.5	0.27
	CD19^+^ cells (%)	11.2 ± 3.0	11.9 ± 3.8	0.76

IR, insulin resistance; non-IR, none insulin resistance; HOMA-IR, homeostasis model assessment for insulin resistance; HOMA-β, homeostasis model assessment of β cell function; DI, disposition index, representing an adjusted insulin sensitivity; TCH, total cholesterol, TG, triglyceride; LDL, low density lipoprotein-cholesterol; HDL, high density lipoprotein-cholesterol; HCY, homocysteine; TSH, thyrotropin; FT3, free triiodothyronin; and FT4, free thyroxine. *Significant difference between the two groups.

### Association Between TG and Insulin Resistance or Insulin Sensitivity

**Figure 3:**
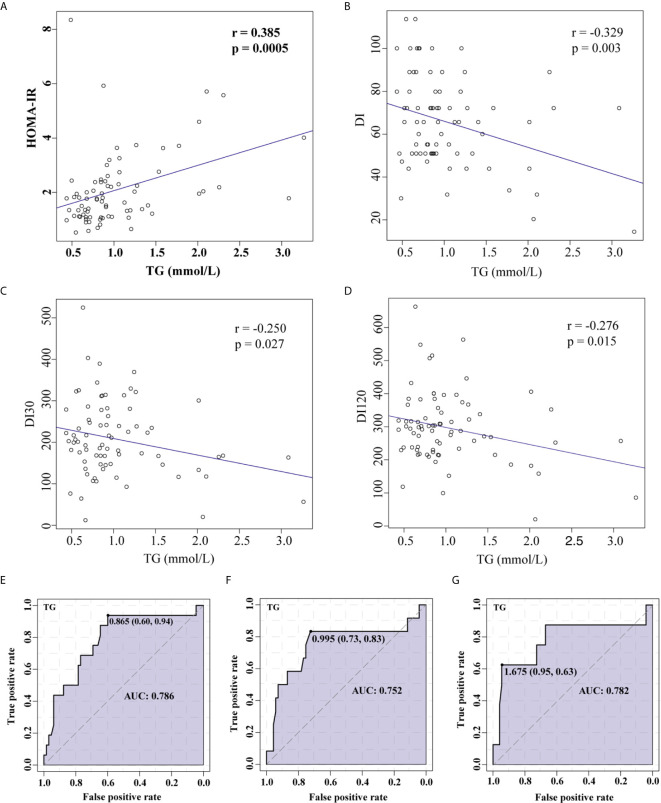
Correlation between TG levels and HOMA-IR or insulin sensitivity demonstrated using Pearson’s linear correlation model and ROC curve analysis. Association between TG and HOMA-IR **(A)**, TG and DI **(B)**, TG and DI30 **(C)**, and TG and DI120 **(D)**. ROC curve analysis for TGs and HOMA-IR **(E)**, DI **(F)**, and DI120 **(G)**.TG, Triglyceride; HOMA-IR, homeostasis model assessment for insulin resistance; DI, disposition index, representing adjusted insulin sensitivity according to HOMA-IR.

In the measures of lipid profile, TG levels were significantly elevated in patients with IR (**Table 2**). Pearson linear correlation model revealed a significant positive association between TG and HOMA-IR, suggesting an increased possibility of insulin resistance as TG levels increased (**Figure 3A**). A significant negative association was also identified between TG and DI (**Figure 3B**), DI30 (**Figure 3C**), DI120 (**Figure 3D**), indicating that a high TG level was closely associated with a decrease in the insulin sensitivity. Furthermore, a receiver operating characteristic (ROC) curve analysis indicated that TGs were associated with HOMA-IR (**Figure 3E**), DI (**Figure 3F**), and DI120 (**Figure 3G**). Moreover, a significant negative association was identified between TSH and HOMA-IR, however, no significant association was found between TG and TSH (**Supplementary Figure 1**).

### An Increase in TG Levels Was Positively Associated With Hyperinsulinemia.

Hyperinsulinemia was defined as a fasting insulin ≥15 mU/L, and/or insulin levels at 2 h ≥80 mU/L during OGTT. In total, 25 enrollments were defined as hyperinsulinemia. Multivariate logistic regression analysis was performed to assess the association between TG and hyperinsulinemia in RPL patients. The results demonstrated that increase in TG was positively associated with hyperinsulinemia (*p* = 0.02). After adjusting for confounding factors including age, and BMI, there was a 2.77-fold higher risk of developing hyperinsulinemia with increasing TG levels (*p* = 0.03) (**Table 3**).

**Table 3:** Logistic regression analysis for hyperinsulinemia and CD3^+^CD4^+^/CD3^+^CD8^+^ ratio.

	Models	B	SE	*P*-value	OR (95% CI)
Hyperinsulinemia	Model I				
	TG levels	1.02	0.46	0.02	2.78 (1.14 - 6.78)
	Model II				
	TG levels	1.02	0.46	0.03	2.77 (1.12 - 6.84)
CD3+CD4+/CD3+CD8+	Model I				
	TG levels	1.13	0.47	0.02	3.10 (1.24–7.72)
	Model II				
	TG levels	1.05	0.63	0.04	2.85 (0.83–9.77)

Model I, variables were introduced into the logistic regression model; Model II, adjustment for confounding factors including age, and BMI. BMI, Body mass index; TG, triglyceride; B, regression coefficient; SE, standard error; OR, odds ratio; CI, confidence interval.

## Increased TG Level Was Positively Associated With the Ratio of CD3^+^CD4^+^ to CD3^+^CD8^+^ Lymphocytes

The ratio of CD3^+^CD4^+^ to CD3^+^CD8^+^ lymphocytes (CD3^+^CD4^+^/CD3^+^CD8^+^) was calculated by using the percentages of CD3^+^CD4^+^ and CD3^+^CD8^+^lymphocyte cells. The ratio of < 1.0 was set as decreased group (N = 22) and those with a ratio ≥ 1.0 were normal group (N = 67) (42). Multivariate logistic regression analysis was performed to assess the association between increased levels of TG and CD3^+^CD4^+^/CD3^+^CD8^+^ ratio in RPL patients. The results showed that increase in TG was positively associated with an increased CD3^+^CD4^+^/CD3^+^CD8^+^ ratio (*p* = 0.02). After adjusting for confounding factors including age, and BMI, there was a 2.85-fold increase in the ratio with an increasing TG levels (*p* = 0.04) (**Table 3**).

### Increased Ratio of CD3^+^CD4^+^ to CD3^+^CD8^+^ Lymphocytes Was Negatively Associated With DI30 and DI120

**Figure 4:**
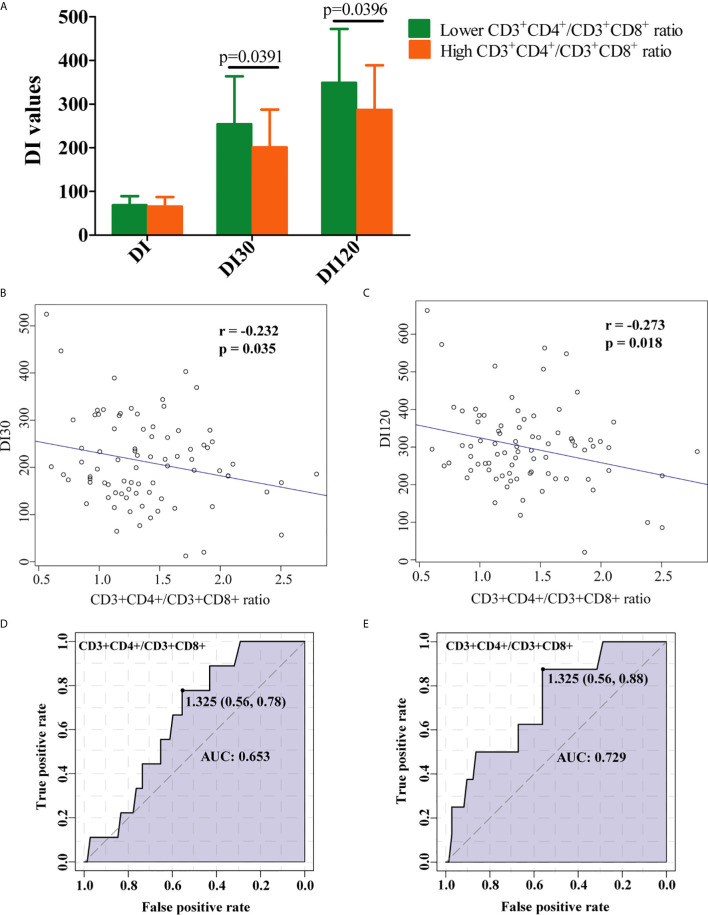
Clinical implication of CD3^+^CD4^+^/CD3^+^CD8^+^ ratio in RPL patients with IR. **(A)** RPL women with decreased CD3^+^CD4^+^/CD3^+^CD8^+^ ratio had significantly higher levels of DI30 and DI120. The increase in the ratio of CD3^+^CD4^+^/CD3^+^CD8^+^ was marked associated with low **(B)** DI30 and **(C)** DI120 levels. ROC curve analysis for CD3^+^CD4^+^/CD3^+^CD8^+^ ratio and DI30 **(D)**, and DI120 **(E)**. Data is presented as mean ± standard deviation. DI, disposition index, representing adjusted insulin sensitivity according to HOMA-IR.

The RPL women with a lower CD3^+^CD4^+^/CD3^+^CD8^+^ ratio of < 1.0 were set as decreased group and those with a ratio ≥ 1.0 were normal group. As presented in **Figure 4A**, DI30 and DI120 were significantly decreased in the high CD3^+^CD4^+^/CD3^+^CD8^+^ group, and the ratio of CD3^+^CD4^+^/CD3^+^CD8^+^ was significantly negatively correlated with both DI30 (**Figure 4B**) and DI120 (**Figure 4C**). Moreover, ROC analysis indicated that CD3^+^CD4^+^/CD3^+^CD8^+^ were associated with DI30 (**Figure 4D**), and DI120 (**Figure 4E**), suggesting that the increased ratio of CD3^+^CD4^+^/CD3^+^CD8^+^ is accompanied by the reduced insulin sensitivity.

## Discussion

RPL has complex pathogenic mechanisms. Using a unique cohort of RPL patients without PCOS and obesity, we found increased TG levels in subjects with IR compared to those without IR. Moreover, the increased TG was positively associated with the ratio of CD3^+^CD4^+^/CD3^+^CD8^+^, and was accompanied by reduced insulin sensitivity and hyperinsulinemia. Thus, our study suggests that elevated TG and altered immune response are associated with insulin resistance in this cohort of RPL patients. Strategies to reduce the elevated TG and/or modulate immune system could be considered for treatments of these patients.

Insulin resistance is a condition in which the body fails to properly use insulin to regulate glucose metabolism. The accumulation of excessive insulin in the blood, described as hyperinsulinemia, with normal blood glucose levels indicates the presence of IR (43–45). Patients with PCOS and/or obese appear to have a higher prevalence of IR, and increased rate of pregnancy loss. Recently, it also has been reported that impaired insulin clearance and hyperinsulinemia could occur in non-obese Asians (20). However, the underlying correlative mechanisms between IR and RPL in patients without PCOS/obese remain inconclusive. To investigate the underlying mechanisms, we recruited a unique cohort of RPL patients without PCOS, suspected PCOS, and obese. The current study revealed that the glucose remained normal levels, however, the insulin levels at corresponding time points were significantly higher during OGTT in the IR group. Additionally, increased early-phase insulin release, total insulin release and lower insulin sensitivity indicated the occurrence of IR. Notably, the diagnose of IR in this study was determined based on a Chinese clinical expert consensus (46), which might be better representation for RPL patients in China. These appearance makes it more consistence to investigate the underlying mechanism between IR and RPL patients without PCOS and obese.

There is evidence that the higher fasting levels of TG are associated with IR (47, 48). These findings are consistent with the results of the present study. In this study, we found that higher TG levels were positively correlated with the risk of hyperinsulinemia in RPL women with and without adjustment of age and BMI. However, the underlying connection between higher TG levels and RPL patients with IR remained elusive. It has been demonstrated in mice that lymphocytes participate in the pathogenesis of IR (49). Yan et al. found that a far greater proportion of newly diagnosed RPL patients with IR had an aberrant CD3^+^CD4^+^, and CD56^+^CD16^+^ cell population compared to RPL patients without IR (34). Our study found that an increase in TG levels positively associated with an increased CD3^+^CD4^+^/CD3^+^CD8^+^ ratio. Furthermore, an inverse correlation between the CD3^+^CD4^+^/CD3^+^CD8^+^ ratio and DI values, reflecting insulin sensitivity were found, and increased CD3^+^CD4^+^/CD3^+^CD8^+^ is an indicator for hyperinsulinemia, revealing the involvement of CD3^+^CD4^+^/CD3^+^CD8^+^ in RPL patients with IR.

Previous studies indicated that an increased ratio of CD4^+^/CD8^+^ was positively correlated with tumor necrosis factor-α (TNF-α) level (50, 51). Notably, TG levels induced a high levels of plasma TNF-α by the increased production of very low density lipoprotein (VLDL) particles (52). And increased TNF-α expression contribute to decreased adiponectin, which plays as an important modulator of insulin sensitivity *via* the downregulation of the peroxisome proliferator activated receptor-α (PPAR-α) (53, 54). From our observations, we postulate that high TG levels induce IR in RPL patients *via* the elevated CD3^+^CD4^+^/CD3^+^CD8^+^ ratio. One possible hypothesis is elevated TG induce aberrant lymphocyte subsets, including increased CD3^+^CD4^+^/CD3^+^CD8^+^, which serves a proinflammatory role by producing TNF-α, leading to reduced adiponectin production and blocking activation of PPAR-α, thereby resulting in decreased insulin sensitivity. All these findings indicate that higher TG levels are associated with the development of IR.

The strengths of this study include: (i) thoroughly consideration of the exclusion criteria for common causes of pregnancy loss such as uterine abnormalities, karyotype abnormality, luteal phase defect, and abnormal antibodies; (ii) an RPL cohort with and without IR independent of PCOS and abnormal BMI. Several limitations were associated with our study: (i) some information related to IR was not obtained, such as the free fatty acid levels, the detail of regular physical exercise, and family history of diabetes; (ii) this study was a cross-sectional design that made it difficult to explore the causal relationship between TG, CD3^+^CD4^+^/CD3^+^CD8^+^ ratio and IR. (iii) there is lack of racial diversity in the studied cohort. It was reported that ethnic differences in TG levels should be considered for identifying insulin resistance (55). Tamura et al., reported that impaired insulin clearance and hyperinsulinemia could occur in non-obese Asians and decreased insulin sensitivity in muscle was well correlated with elevated TG levels in non-obese Asians (20). It is possible that TG can be used as an indicator of IR in RPL patients with racial and ethnic disparities. Finally, future studies with diverse populations and large survey samples are warranted.

In conclusion, the results of the present study demonstrated significant increases in the early phase insulin secretion and total insulin secretion during OGTT and an increased TG levels in RPL patients with IR. The increased serum level of TG was positively associated with postprandial hyperinsulinemia. The role of CD3^+^CD4^+^/CD3^+^CD8^+^ ratio in insulin resistance contributes to the associated interference with islet compensation function. Further investigation is required to determine if CD3^+^CD4^+^/CD3^+^CD8^+^ ratio is able to act as immune indicators of insulin resistance and islet β-cell function in RPL patients.

## Data Availability Statement

The raw data supporting the conclusions of this article will be made available by the authors, without undue reservation.

## Ethics Statement

The studies involving human participants were reviewed and approved by the Ethics Committees of Shanghai First Maternity and Infant Hospital. The patients/participants provided their written informed consent to participate in this study.

## Author Contributions

LF and YL conceived and designed the experiments. MD, YG, and YL performed sample collection. YL performed experimental processing of the samples and analyzed the data. YL wrote the manuscript, and LF revised the manuscript. SB, LF, and JZ contributed to discussions and reviewed the paper, and gave their approval to the final version of the manuscript. All authors contributed to the article and approved the submitted version.

## Funding

This work was supported by the National Natural Science Foundation of China (81903325), and the China Postdoctoral Science Foundation (2018M632138).

## Conflict of Interest

The authors declare that the research was conducted in the absence of any commercial or financial relationships that could be construed as a potential conflict of interest.

## Supplementary Material

Supplementary Figure 1 Correlation analyses between lipid profiles, the lymphocyte subsets and insulin resistance. HOMA-IR, homeostasis model assessment for insulin resistance; HOMA-β, homeostasis model assessment of β cell function; ISI_M_, Matsuda insulin sensitivity index; DI, disposition index, representing an adjusted insulin sensitivity; TCH, total cholesterol; TG, triglyceride; TSH, thyrotropin; * indicates *p* < 0.05, ** indicates *p* < 0.01, *** indicates *p* < 0.0001.
