# Supplementary figures and images for: Triglyceride Induced Metabolic Inflammation: Potential Connection of Insulin Resistance and Recurrent Pregnancy Loss
Source: Front Endocrinol (Lausanne). 2021 Apr 15;12:621845. doi: 10.3389/fendo.2021.621845 (PMC8082681; doi:10.3389/fendo.2021.621845)

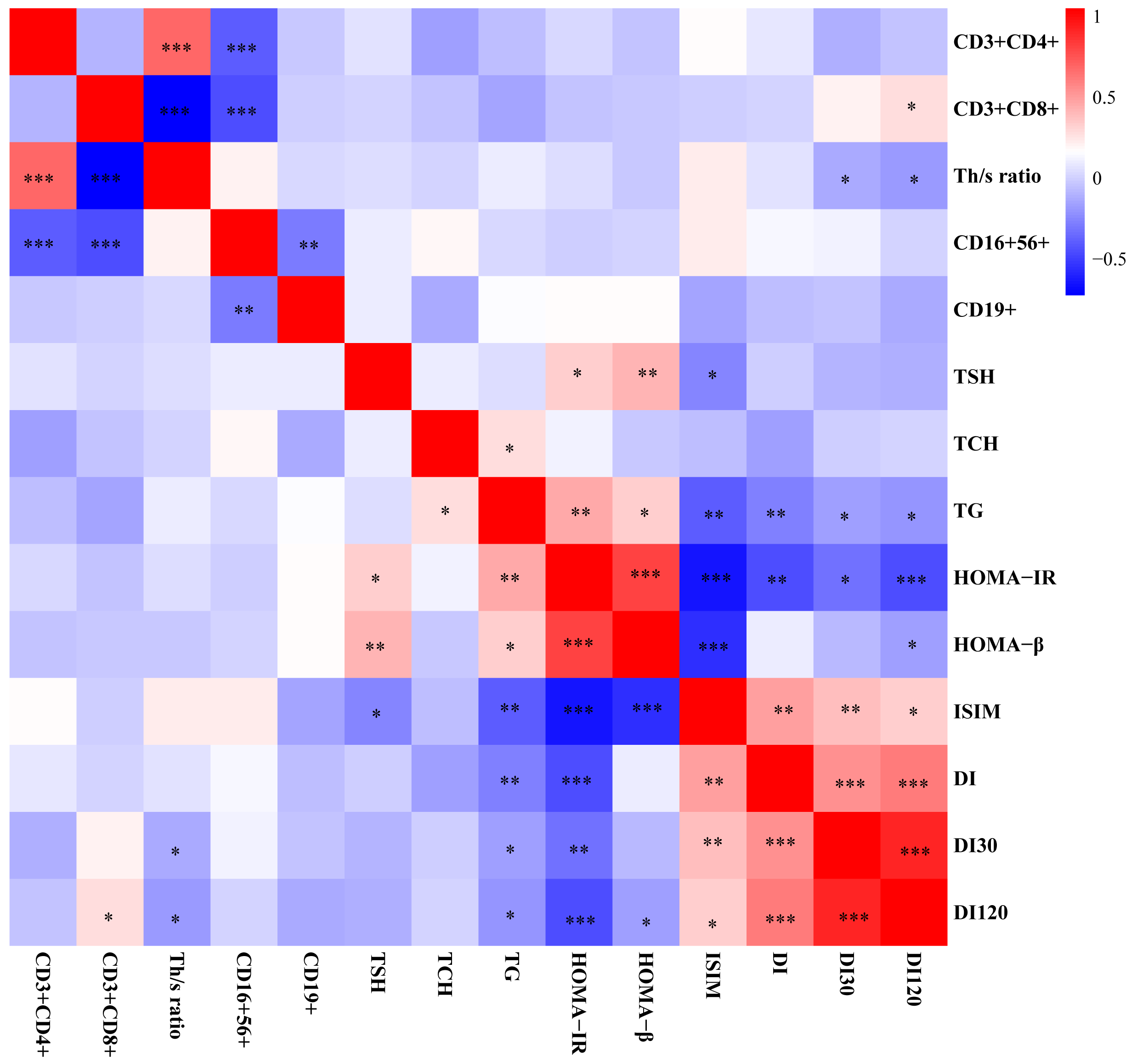

Supplement: Supplementary Figure 1 — Correlation analyses between lipid profiles, the lymphocyte subsets and insulin resistance. HOMA-IR, homeostasis model assessment for insulin resistance; HOMA-β, homeostasis model assessment of β cell function; ISIM, Matsuda insulin sensitivity index; DI, disposition index, representing an adjusted insulin sensitivity; TCH, total cholesterol; TG, triglyceride; TSH, thyrotropin; * indicates p < 0.05, ** indicates p < 0.01, *** indicates p < 0.0001. [file Image_1.tif]
